# Extracellular Vesicles in CNS Developmental Disorders

**DOI:** 10.3390/ijms21249428

**Published:** 2020-12-11

**Authors:** Ana Rita Gomes, Nasim Bahram Sangani, Tiago G. Fernandes, M. Margarida Diogo, Leopold M. G. Curfs, Chris P. Reutelingsperger

**Affiliations:** 1 Department of Bioengineering and IBB—Institute for Bioengineering and Biosciences, Instituto Superior Técnico, Universidade de Lisboa, 1049-001 Lisboa, Portugal; ana.b.gomes@tecnico.ulisboa.pt (A.R.G.); tfernandes@tecnico.ulisboa.pt (T.G.F.); margarida.diogo@tecnico.ulisboa.pt (M.M.D.); 2 Instituto de Medicina Molecular João Lobo Antunes, Faculdade de Medicina da Universidade de Lisboa, 1649-028 Lisboa, Portugal; 3 Department of Biochemistry, Maastricht University, Cardiovascular Research Institute Maastricht, 6200 MD Maastricht, The Netherlands; nasim.sangani@maastrichtuniversity.nl; 4 GKC-Rett Expertise Centre, Maastricht University Medical Centre, 6229 ER Maastricht, The Netherlands; leopold.curfs@maastrichtuniversity.nl

**Keywords:** neurodevelopmental disorders, extracellular vesicles, exosomes, microvesicles, CNS, neurons, astrocytes, glia

## Abstract

The central nervous system (CNS) is the most complex structure in the body, consisting of multiple cell types with distinct morphology and function. Development of the neuronal circuit and its function rely on a continuous crosstalk between neurons and non-neural cells. It has been widely accepted that extracellular vesicles (EVs), mainly exosomes, are effective entities responsible for intercellular CNS communication. They contain membrane and cytoplasmic proteins, lipids, non-coding RNAs, microRNAs and mRNAs. Their cargo modulates gene and protein expression in recipient cells. Several lines of evidence indicate that EVs play a role in modifying signal transduction with subsequent physiological changes in neurogenesis, gliogenesis, synaptogenesis and network circuit formation and activity, as well as synaptic pruning and myelination. Several studies demonstrate that neural and non-neural EVs play an important role in physiological and pathological neurodevelopment. The present review discusses the role of EVs in various neurodevelopmental disorders and the prospects of using EVs as disease biomarkers and therapeutics.

## 1. Introduction

### 1.1. Extracellular Vesicles

Cell-to-cell communication is a fundamental process in coordinating the functions and interactions between the diverse neural cell populations in the central nervous system (CNS) and is mainly organized through secretion of molecules in the intercellular space [1]. Extracellular vesicles (EVs) have been recognized as communication vehicles playing an important role in neural cell proliferation and differentiation, and also in immune modulation and senescence [2].

EVs can be classified and distinguished according to their biogenesis, sub-cellular origin, cargo, size and method of isolation.

A subset of EVs, the exosomes, originate from the inward budding of endosomal membranes, giving rise to the formation of multivesicular bodies (MVBs). MVBs typically depict a diameter between 250–1000 nm and contain intraluminal vesicles (ILVs), which are released into the extracellular space as exosomes after the fusion of MVBs with the plasma membrane [3]. Exosomes are the smallest EVs and range from 30 to 100 nm in diameter [4]. The microvesicles (MVs) form another subset of EVs. They are larger than exosomes, with a diameter between 0.1 and 1µm. MVs are released from cells by plasma membrane budding [5]. The largest subset of EVs are the apoptotic bodies, which are shed from a dying cell executing apoptosis [6]. The apoptotic bodies can vary in size between 1 and 5 µm in diameter.

EVs have been isolated from a great variety of fluids, including supernatants of cultured cells, blood, urine, cerebrospinal fluid (CSF) and serum [7]. Isolation of the different EV subtypes has been accomplished using several methods, such as isolation by size, immunoaffinity capture or precipitation. Isolation by differential ultracentrifugation is widely considered the gold standard method [8,9,10]. It should be noted, however, that physical and molecular overlap between the EV subsets has precluded the definition of specific EV subtype marker proteins to date [11].

### 1.2. Molecular Composition of EVs

EVs carry a diverse set of molecules that can be transported over short and long distances to recipient cells. There, they execute defined biological functions, which contribute to health and disease. The composition of EVs is determined by their biogenetic pathway and the microenvironment of the parental cell [12]. The composition may also contribute as a fingerprint for establishing the origin and type of EVs, which is relevant if EVs are to be considered as biomarkers. However, this is not as unambiguous as suggested by many papers on EV research. The endosomal sorting complex required for transport (ESCRT) and accessory proteins are necessary for MVB biogenesis; hence, ESCRT proteins and Alix and TSG101 are considered standard markers of exosomes, regardless of the parental cell type [13]. It has been shown that cells depleted of the ESCRT machinery are still able to produce CD63-positive exosomes by utilizing the sphingomyelinase—ceramide machinery [14]. A recent study with exosomes extracted from neural progenitor cells (NPCs) derived from human induced pluripotent stem cells (hiPSCs) expressed lower levels of Alix, TSG101, Hsp70 and also CD63, in comparison with hiPSC-derived cardiac cells [15]. This might suggest a different protein machinery for neural derived-exosome biogenesis and tracking (i.e., ESCRT-independent pathways) [13].

Other membrane proteins commonly found in exosomes and enriched when compared with cell lysate content are integrins and tetraspanins (such as CD9, CD81, CD82, CD63 and CD37). Exosomes also contain cytosolic proteins, such as heat-shock proteins (Hsp70, Hsp90), in addition to cytoskeletal proteins, like tubulin and actin. Moreover, exosomes contain small GTPases, such as RAB27A, RAB11 and RAB35, which play an important role in intracellular trafficking in secretory pathways during vesicle formation and also in exosome release [16].

Besides the abovementioned typical protein cargo, primary cortical neuron-derived exosomes have been characterized and identified with synaptic proteins, such as L1 cell adhesion molecule (L1CAM), glycosylphosphatidylinositol (GPI)-anchored prion protein and glutamate receptor subunit GluR2/3 [17]. EV types carrying specific neuronal protein cargo will be discussed further in the following sections.

Comprehensive analyses of the composition of EV subtypes derived from different cell types revealed a substantial difference in lipidomics and proteomics between exosomes and MVs [17]. MVs encompass more proteasomes, and endoplasmic reticulum and mitochondrial proteins, whereas exosomes express relatively more proteins that function at the interface with the environment [18]. The lipid content also differs between MVs and exosomes. MVs are enriched in ceramides and sphingomyelins, and exosomes carry more glycolipids and free fatty acids [19]. Interestingly, apoptotic bodies have a content that resembles those of cell lysates, yet they also express unique features within their cargo, such as enrichment of thioredoxin peroxidase II, Alix, 14-3-3 and galectin-3 [20]. Apoptotic bodies express more specific surface markers such as phoshatidylserine (annexin A5-binding), thrombospondin and C3b [7,21].

EV types also carry a wide range of genetic material including DNA, mitochondrial DNA (mtDNA), and coding and non-coding RNAs (long non-coding RNAs, micro (mi)RNAs and circular RNAs) [22].

Experimental evidence has demonstrated that the genetic information transferred by EVs can be used by the transcriptional and translational machineries of the recipient cell [22]. Morel et al. identified miR-124a to be abundantly expressed by neuronal exosomes and demonstrated, both in vivo and in vitro, that the neuronal exosomes transfer miR-124a to astrocytes, which consequently upregulated the expression of GLT1 [23].

In a more recent breakthrough, Men et al. have demonstrated that the miRNA profile of secreted exosomes is different from the one observed in live neuronal cells [24]. By generating a cell-type-specific ILVs/exosome reporter (CD63-GFPf/f) in mice, the authors observed that an undescribed neuron-specific miRNA, miR-124-3p, was internalized into astrocytes, also upregulating the glutamate transporter GLT1 [24].

Interestingly, the aforementioned study using vesicles isolated from hiPSC-derived cells from distinct lineage, revealed differential expression of the analyzed miRNA, suggesting the lineage specificity of miRNA cargo. For example, miR-34a-3p was observed to be highly expressed in the hiPSC-derived NPCs, in addition to miR-133a and miR-133b, which may be involved in neurite growth [15]. Nonetheless, miRNAs observed in exosomes from non-neuronal cells, such as mesenchymal stem cells (MSCs), could function as promotors of neurogenesis and neurite remodeling, similar to miR-133b [25]. A significant number of studies have shown the potential of exosomal miRNA as biomarkers, both for diagnostic purposes and for studying several neurodevelopmental and neurodegenerative disorders [26]. A study using genome-wide next-generation sequencing revealed substantial differences in exosomal miRNA profiles between CSF and serum when compared with miRNA found in the brain [27]. Half of the miRNAs already reported in the brain were only found in CSF exosomal fractions. Particularly, miR-1911-5p was detected in both brain tissue and CSF. Therefore, brain pathophysiology could be inferred by the analysis of exosomal pathogenic proteins and miRNA extracted from CSF and other biological fluids. As we will discuss next, EVs, predominantly exosomes, could also provide novel mechanisms of intercellular communication during nervous system development, offering new clues on the progression of neurodevelopmental pathologies.

## 2. EVs Mediate Communication in CNS—During and Post-Development

Several studies have demonstrated that the various EV types play a role in mediating critical interactions during CNS development, mainly in cellular connection and circuit maintenance.

A study by Marzesco et al. was one of the first reports describing the existence of EVs during neurodevelopment. The vesicles positive for the stem cell marker prominin-1 (CD133) were found in the luminal fluid of the neural tube in embryonic mouse brains [28]. Moreover, it was also observed that primary cultures of cortical neurons were able to secrete exosomes containing specific neural proteins [17]. Similarly, mature cortical and hippocampal neurons also secrete exosomes [29]. These studies highlight the role of EVs in regulating synaptic activity during development, particularly their role in neuronal communication mediated by glutamatergic synaptic activity, 2-amino-3-(3-hydroxy-5-methyl-isoxazol-4-yl) propanoic acid (AMPA) and N-methyl-d-aspartate (NMDA) receptors [29].

In the specific case of neurons, exosomes are released from post-synaptic soma or dendrites [24], and they mediate several processes, such as the maintenance of homeostasis by triggering synapse pruning by microglial cells [30], or the outflow of molecular information to neighboring cells, mediated by miRNAs. These miRNAs may induce gene expression in recipient cells in an activity-dependent manner [31]; as previously described, miR-124 internalized by astrocytes is capable of regulating the glutamate transporter (GLT1) levels, as well as glutamate uptake in the brain [23].

Several reports also describe the release of EVs, mainly exosomes types, by astrocytes during brain development under normal neuronal activity or during oxidative stress or other stressful insult conditions. Neuroactive substances, like Hsp70 [32] or synapsin I, are released from non-neural cells via exosomes and ultimately promote neurite outgrowth and neuronal survival [33]. Oligodendrocytes, responsible for myelin sheath production, have been demonstrated to release exosomes with myelin proteins, which could be triggered by glutamate release from active neurons. Moreover, these oligodendrocyte secreted-exosomes are responsible for increasing the firing rate activity of neurons [34].

Finally, microglia, the immune cells present in the brain, play a relevant role in immune regulation, mainly by propagating vesicles with cytokines, chemokines and reactive oxygen species, which will mediate the inflammatory response. For example, stimulation of cortical microglia with lipopolysaccharides (LPS) resulted in the release of exosomes with an enriched cargo of pro-inflammatory cytokines, such as IL-1β, which are responsible for the propagation of inflammation [35]. Microglia can also release immunomodulatory exosomes containing major histocompatibility complex (MHC) Class II receptors. Furthermore, another study proved that microglia-derived MVs are responsible for stimulating synaptic vesicle release in presynaptic terminals, by involving neuronal sphingosine and ceramide production [36]. Another study showed that stimulators of serotonin (5-HT) receptors increased microglial exosome release and that exosomes enclose an insulin-degrading enzyme that is known to ultimately degrade the neurotoxic peptide amyloid-β [37]. Actually, this study aimed to demonstrate the functional signaling between neurons (serotonergic neurons) and microglia.

**Figure 1:**
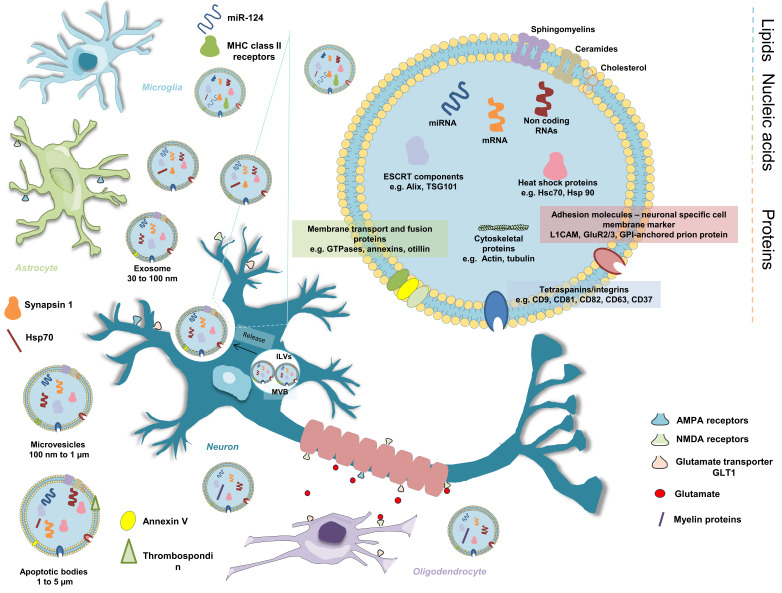
Schematic illustration of extracellular vesicles (EVs) in cell–cell communication in the CNS. EVs mediate intercellular communication between neurons, oligodendrocytes, astrocytes and glial cells. The EV is composed of lipids, proteins and polynucleotides. The cellular origin of EVs will define the cargo content and signaling capacity. For example, neural-derived exosomes carry synaptic cell adhesion molecules: neuronal-specific cell membrane marker L1CAM, GPI-anchored prion protein and GluR2/3. Proteins involved in vesicular trafficking, such as Rab proteins, annexins and cytoskeletal proteins, are present in EVs derived from neuronal and non-neuronal cells.

Taken together, these reports show the important role of EVs, mainly exosomes, in CNS development, in further modulation of neuronal activity, and cell-to-cell communication (Figure 1), neuroprotection and repair.

## 3. EVs in Developmental Pathology of the Nervous System

Alterations in brain-derived EV composition (Table 1) have been associated with changes in the crosstalk between neural cells, some of which have been linked to neurodegenerative disorders. Given their role in mediating intercellular communication at different stages of normal and pathological CNS development [2], including neural cell proliferation and differentiation, synaptic formation, and learning and memory processes [38], neurodevelopmental pathologies will benefit from in-depth knowledge about EVs in the CNS.

**Figure 2:**
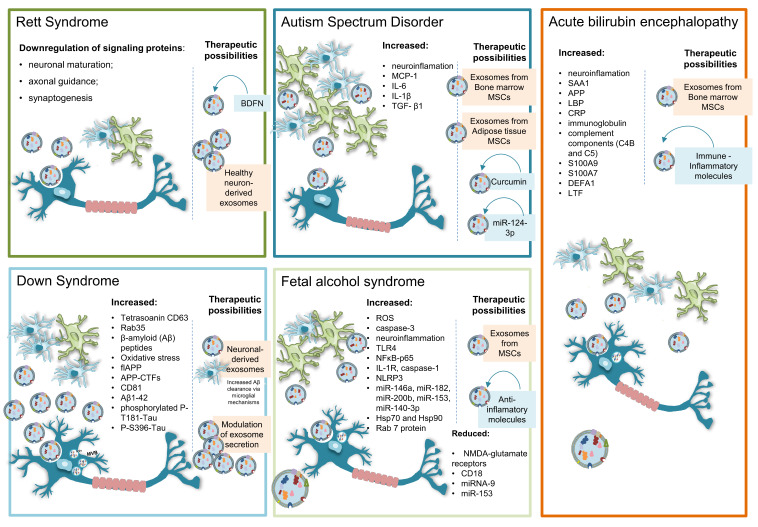
Schematic illustration summarizing the main changes observed in the cargo of EVs in the neurodevelopmental disorders that are discussed in this review. Therapeutic possibilities using or targeting exosomes are also indicated.

EVs are projected to offer novel therapeutic avenues (Table 2) to treat CNS diseases and play a role as biomarkers of disease status and progression (Table 3). The analysis of EVs’ molecular signals such as mRNA, miRNA, lipid or protein content, and their correlation with human brain developmental pathologies will be discussed next and summarized in Figure 2. In the following sections, the terminology of EV subtypes is in accordance with the original work.

### 3.1. Rett Syndrome

Rett syndrome (RTT) is a severe neurological disorder affecting brain development and function and is caused by mutations in the gene encoding the methyl-CpG-binding protein 2 (MeCP2), localized in the X chromosome [55]. MeCP2 mutations lead to developmental regression, with a range of neurodevelopmental defects, including loss of speech, acquired movement skills and severe cognitive impairment after an apparently normal development [56]. In vitro studies using RTT hiPSC-derived neurons showed impaired neuronal maturation, supported by the presence of fewer synapses, smaller soma size, altered calcium signaling, functional defects in firing activity and excitatory/inhibitory (E/I) imbalance [57,58]. These phenotypic alterations observed in RTT could also be closely related to changes in the biological content of exosomes, as discussed next.

Recently, Sharma et al. used neurons derived from hiPSCs lacking MeCP2 to analyze the effect of MeCP2 deficiency on the protein content of exosomes [39]. This study showed that the loss of MeCP2 function (MeCP2LOF) was associated with a dramatic change in the protein content of exosomes and the signaling bioactivity of the exosomes. Proteomic analysis revealed that some neurodevelopmental signaling proteins, mainly associated with neuronal maturation, axonal guidance and synaptogenesis, were upregulated in isogenic control (IC) exosomes when compared with MeCP2LOF. Neuronal RTT cultures were then treated with healthy exosomes, which increased puncta densities (Synapsin1 staining), resulting in an increase in synaptogenesis. Furthermore, spike recordings revealed an improvement of neuronal activity with higher network synchronization. In this context, exosomes displayed a prominent role in regulating important molecular pathways. The involvement of RNA, miRNA and circRNA needs further investigation.

Other experimental models of RTT revealed impairments in the length and type of dendritic spines causing abnormalities in synaptic communication. A study with a Mecp2-deficient male mice showed thalamo-cortical axon arbor failure, resulting in reduced complexity and density of the dendritic branches in neurons [59]. Another study using 3D forebrain organoids derived from RTT hiPSCs demonstrated a decrease in the number of more mature branched spines and an altered electrophysiological profile characterized by defects in spontaneous synaptic transmission and connection [60]. It has been hypothesized that synaptic physiology is, at least partially, mediated by exosome release [29], implying that RTT pathology may be associated with aberrant exosome biology. Both in vivo and in vitro models may help to provide a mechanistic understanding of the role of exosomes in RTT pathology of the different brain regions.

In addition, exosomes were revealed to be potential agents for translational research, presenting themselves as treatment options for targeting pathological features of RTT, particularly synaptic activity regulation.

Strong evidence suggests that brain-derived neurotrophic factor (BDNF) is significantly reduced in the brains of RTT patients [61] and RTT mouse models [62]. MeCP2 mutations affect BDNF gene transcription, mRNA translation and protein trafficking, contributing to the RTT symptomatology. BDNF binds to a specific membrane-bound receptor, tropomyosin-related kinase B (TrkB), organizing signaling cascades that modulate neuronal differentiation, survival in early development and synaptic transmission [63]. A promising diagnosis approach could rely on EV isolation from the peripheral blood of RTT patients. In a study by Suire et al., it was reported that adults with aging-associated walking speed decline showed higher levels of proBDNF and BDNF in isolated EVs, specifically an enriched subpopulation of neuronal origin, expressing the neuronal marker L1CAM [64]. In addition, mRNA levels of BDNF transcripts were observed to be lower in brain samples from RTT patients. Thus, the identification and quantification of specific miRNAs present in circulating brain-derived EVs could contribute to the diagnosis and also to reveal important cues about the affected pathways and mechanisms associated with the pathology [63]. BDNF overexpression in hippocampal neurons was shown to rescue several RTT-associated phenotypes and dendritic atrophy [62]. However, the use of the natural form of this neurotrophic factor is not a useful clinical approach due to its short half-life and inability to cross the blood–brain barrier (BBB) [62]. Nevertheless, understanding the role of exosomes in RTT can open therapeutic avenues based on exosomes as carriers of therapeutic molecules; for example, BDNF or miRNAs that regulate BDNF expression [63].

### 3.2. Autism Spectrum Disorder

The autism spectrum disorder (ASD) is a lifelong neurodevelopmental pathology with core abnormalities in social and communication capacity, and stereotyped behaviors and interests [65]. Brain development defects present in ASD are associated with disruption of cell proliferation and differentiation, neuronal maturation, neurite outgrowth, altered synaptogenesis and reduced neural network functionality [65,66,67]. Indeed, inflammation in the CNS and neuro-immune crosstalk dysregulation are prevalent in ASD patients. The described neural dysfunction could be directly related to neuroinflammation, which is characterized by the activity and proliferation of glial cells, astrocytes and microglia [68]. Robust studies have shown that microglia and astrocytes can release exosomes with pro-inflammatory mediators, such as cytokines, chemokines, reactive oxygen species (ROS) and secondary messengers that are important players in the inflammatory process [69]. In human brain samples from ASD patients, microglial and astroglial activation was found to be increased, particularly in the cerebellum. More importantly, the upregulation of macrophage chemoattractant protein-1, MCP-1 and pro-inflammatory interleukin IL-6 were observed in high amounts in reactive astrocytes in the cerebellum and the cortical and subcortical white matter regions. The anti-inflammatory cytokine tumor growth factor-β1 (TGF-β1) was also strongly increased, not only in neuroglia but also in the granular cell layer and Purkinje cell population, presenting significant degeneration [70].

A recent study reported that EVs isolated from the serum of ASD patients have a significant increase in total protein concentration when compared with healthy controls [40]. Moreover, when ASD serum-derived EVs were cultured with human microglia cells, secretion of IL-1β, a pro-inflammatory cytokine, was observed. The same authors previously observed an increased amount of mtDNA in EVs from the serum of ASD patients. They suggested that EV mtDNA may trigger the pro-inflammatory response of immune cells and may partially explain the immune dysregulation reported in autistic patients [71].

The current literature strongly points towards a role of EVs in modulating the pro-inflammatory responses of microglia and astrocytes, and neuroinflammation. Most studies have been performed with non-ASD animal models but the results of these studies can also have significance for understanding ASD pathology and for designing therapies to alleviate ASD disease features. A recent study explored the role of bone marrow MSC exosomes in LPS-induced neuroinflammation in the cerebellum of rats. MSC exosomes triggered and stimulated the release of pro-inflammatory cytokines, such as tumor necrosis factor-α and interleukins IL-1β and IL-6, by the activated astrocytes and microglia, causing neuronal cell degeneration and synaptic dysfunction. However, in addition to this paracrine pro-inflammatory activity, the exosomes exhibited a protective effect, predominantly on Purkinje neurons, as shown by a reduction in DNA damage and apoptosis [72].

Plasma levels of the endocannabinoid anandamide, produced in post-synaptic cell membranes, were shown to be reduced in ASD patients [73]. Thus, the lack of binding of anandamide to the receptor CB1 might be implicated in sustained presynaptic transmission of target GABAergic neurons in ASD. Interestingly, another study demonstrated that microglial EVs carrying anandamide on their surface promote a significant decrease in inhibitory postsynaptic currents of neurons [74]. Consequently, the use of healthy derived microglial EVs could potentially be a therapeutic strategy to restore the excitation/inhibition balance in ASD.

The loading of the exosomes with specific cargos that will interfere with the host cells, such as miR-124-3p, could also alleviate the phenomenon of neuroinflammation. Recent work disclosed that miRNA-124-3p from microglial exosomes was responsible for suppressing mTOR signaling, thus inhibiting neuroinflammation, consequently improving the neurologic outcome by promoting neurite outgrowth [75]. This concept of customized exosome packaging was previously tested in vivo by encapsulating curcumin. The exosomes containing curcumin were delivered intranasally to an LPS mouse model and afforded protection against inflammation, with reduced levels of interleukin IL-1β being produced by CD45.2 microglial cells [76].

Recently, a black and tan brachyury (BTBR) mouse model (a model with autistic-like behaviors and all the core symptoms of ASD) was used in an in vivo study for intranasal administration of exosomes secreted by MSCs [50]. Administration of MSC exosomes increased social interactions and reduced repetitive behaviors. RNA sequencing revealed upregulation of miRNAs such as miRNA-143, possibly related to the immunomodulatory effect of MSC exosomes. The same authors recently published a preclinical study in which exosomes extracted from adipose-derived MSCs were administered intranasally and intravenously to BTBR and Shank3 mutated mice [51]. The disruption of the gene Shank3 is associated with some ASD features, such as cognitive and motor impairments. In both animal models, the ASD behavioral phenotype was improved, mainly by using non-invasive intranasal administration. The same authors performed RNA sequencing and proteomics to determine the effects of MSC exosomes in cultured primary neurons [52]. They observed the upregulation of proteins related with anti-inflammatory processes and with immunomodulation. Interestingly, BDNF was amongst the upregulated growth factors, suggesting a role for BDNF as a mediator of neuroprotection and neurogenesis.

More in-depth studies are needed to reveal the cause–consequence relationships between the molecular and biological cues extracted from EVs (cytokines, pro-inflammatory molecules, misfolded proteins, miRNAs) and ASD pathology. Such studies could be performed with hiPSCs derived from ASD patients. The hiPSCs can be differentiated into cortical [77] and cerebellum organoids [78], offering the possibility to study regional aspects of pathogenesis. This in vitro approach could provide important clues for understanding the mechanisms of neuroinflammation that are responsible for the neuronal disruption observed in ASD. It can also provide more accurate knowledge about the (therapeutic) role of EVs in ASD.

### 3.3. Down Syndrome

Down syndrome (DS) is a human genetic disease caused by trisomy of chromosome 21 (Hsa21). This pathology is characterized by early developmental brain abnormalities; early onset of Alzheimer’s disease (AD) is frequently observed as well [79,80]. The early phenotype of this pathology includes enlarged endosomes and the associated dysfunctional pathways in neurons, which could be correlated with brain developmental abnormalities and intellectual disabilities [81].

Recent studies have demonstrated that exosome secretion was increased almost 40% in post mortem brain tissue isolated from individuals with DS, when compared with healthy controls [82]. A trisomy (Ts2) murine model of DS and human DS fibroblasts grown in vitro were also used to study enhanced exosome secretion, supporting the theory that they function as a disposal mechanism to relieve accumulated endosomal abnormalities. Exosome release in the DS brain was influenced by enhanced transcription of tetraspanin CD63 and enhanced protein expression of Rab35, both regulators of exosomes biogenesis. In fact, CD63 knockdown in fibroblasts from DS patients significantly increased the number of intracellular endosomes, in addition to a significant reduction of exosome release [82]. In a more recent work using a Ts2 mouse model, it was observed that the higher levels of exosomes released in DS models was associated with more MVBs (fully matured late endosomes) per neuron and an increased number of ILVs per MVB when compared with controls. Here again, neuronal exosomes were demonstrated to act as vehicles for neurotoxic material release and to serve as protection for neurons against chronic endosomal dysfunction [41].

As described for AD pathology, individuals with DS also exhibit abnormal accumulation of β-amyloid (Aβ) peptides in neuronal endosomes and in MVBs [83]. The abnormal processing of β-amyloid leads to increased amyloid secretion and, consequently, to increased oxidative stress. Indeed, accumulation and early deposition of β-amyloid in superficial layers of the frontal cortex were observed in DS patients and were associated with the start of early neurodegeneration [84]. This is not surprising since β-amyloid precursor protein (APP) gene, which is responsible for amyloid β production throughout the brain, is triplicated in DS patients because it is localized in Hsa21 [85,86].

More recently, exosomes isolated from DS patients, Ts2 mouse brains and human DS fibroblasts were observed to be enriched in full-length APP (flAPP) and in APP carboxyl-terminal fragments (APP-CTFs) [42]. It was demonstrated that APP is sorted into exosomes, where it is proteolytically cleaved into Aβ peptides [87]. The existence of exosomes enriched with APP-CTFs in DS is controversial. As previously stated, DS neurons expel these neurotoxins from the intracellular space via exosomes to survive. Once in the extracellular space, these exosomes can play a pathogenic role by propagating APP-CTFs’ neurotoxic metabolites into healthy/newborn neuronal cells [42,88].

Blood samples from DS patients revealed increased CD81 levels compared with the non-DS controls [43], indicating more abundant exosome secretion in the DS brain. Moreover, the secreted neuronal exosomes not only contain Aβ peptide products but also hyper-phosphorylated species of Tau (P-Tau). Disturbed Tau phosphorylation has been reported during early fetal development in DS [44]. Another study demonstrated elevated levels of Aβ1-42, phosphorylated P-T181-Tau and P-S396-Tau in circulating exosomes from neuronal origin in individuals with DS at an early age when compared with age-matched controls [45]. The higher P-Tau levels observed in neuronal exosomes in early developmental stages of DS could also provide insights into the early neuropathological developmental defects associated with DS and later on into the development of AD-related symptoms. The aberrant P-Tau levels were explained by deregulation of two genes localized on Hsa21: (i) the dual-specificity tyrosine-phosphorylation-regulated kinase 1A (DYRK1A) gene and (ii) the regulator of calcineurin 1 (RCAN1) gene, both expressed in DS brains and implicated in the dysregulation of Tau phosphorylation [89].

Interestingly, the progressive transmission of Aβ and P-Tau proteins throughout brain cells mediated by exosomes has been recently studied [90]. Exosomes extracted from neuronal cells (hiPSC-derived), expressing the repeat domain of Tau P301L and V337M mutations, were injected into wild-type mouse brains, where they were shown to be the mediators of long-distance propagation of the Tau inclusions that were found to be present throughout the brain, triggering extensive degeneration of neuronal dendrites.

Moreover, a recent study also proved that exosomes produced by hiPSC-derived neurons, expressing mutant Tau (mTau), were capable of in vivo propagation of P-Tau pathology after their injection into mouse brains [91]. Furthermore, the proteome cargo of the mutant exosomes was altered, with exclusive proteins being expressed that could be the ones responsible for the propagation of the pathogenesis, such as an endogenous inhibitor of the PP2A phosphatase (responsible for the regulation of P-Tau phosphorylation).

Neuron-derived exosomes extracted from either plasma or CSF can reveal relevant neuropathological cues about DS progress and predict the inception of AD. On the other hand, the intracranial infusion of neuronal-derived exosomes into the brains of an APP transgenic mouse model increased Aβ clearance via microglial mechanisms [84]. Indeed, the therapeutic enhancement of exosomes for homeostatic secretion of toxic material during the early stages of development of DS may be an advantage. However, it is also important to consider the pathogenic role mediated by the exosomal cargo that is propagated into the naïve neurons. Advances in the modulation of exosome secretion must surpass the mechanistic controversy, such as the upregulation of neural exosome secretion induced by sphingomyelin synthase 2 SMS2 knockdown, a sphingolipid-metabolizing enzyme [92]. This induced system demonstrated that neuronal cells treated with SMS2 siRNA enhanced Aβ uptake into microglial cells, which are then degraded in lysosomal compartments. The authors propose that microglia can take up Aβ more promptly after the excessive production of Aβ in the presence of exosomes, observing the reduction of the extracellular amounts of Aβ in co-cultures of neuronal and microglial cells. In addition, more advances in exosome engineering processes for neuronal targeting and cargo modulation must be combined for increasing the possible therapeutic effectiveness, such as decreasing AD inception in DS patients.

### 3.4. Fetal Alcohol Syndrome

The prenatal exposure to alcohol can cause developmental deficits, termed fetal alcohol spectrum disorders (FASDs), which include growth deficits and neurodevelopmental delay, affecting cognition and behavior [93,94]. Several studies have already shown the molecular and cellular consequences of chronic alcohol exposure during early embryonic development, such as interference in neural progenitor cell proliferation, neuronal migration and differentiation. Furthermore, if exposure to alcohol occurs at stages following cell differentiation, it could result in a reduced number of formed synapses and in neuronal cell death [95].

Chronic alcohol exposure increased ROS generation and caspase-3 activation and directly affected cell death in both the cerebral cortex [96] and cerebellum [97]. The increased levels of caspase-3 in the cerebral cortex have been associated with the inhibition of NMDA-glutamate receptors and the activation of gamma-aminobutyric acid (GABA) receptors. The reduction of NMDA-stimulated Ca^2+^ entry into neonatal neurons during brain development could underlie learning deficits, which would therefore be direct consequences of the toxic and teratogenic effect of alcohol exposure [96].

As verified in experimental studies during embryogenesis, astrocytes and microglia are also affected by ethanol exposure, due to the impairment of the radial glia (RG) progenitor pool and its differentiation into neurons and astrocytes. As a result, synaptic transmission and plasticity are impaired and increased neuroinflammation is observed in both astrocytes and neuronal–glial communications. EVs were reported to be involved in regulating intercellular signaling between glial cells and neurons under ethanol exposure conditions [46]. For these studies, the authors used neurons and astrocytes in culture, with the astrocytes being exposed to ethanol. The EVs extracted from the treated astrocytes increased in number and changed their content with an increase in inflammatory-related proteins, such as TLR4, NFκB-p65, IL-1R, caspase-1 and NLRP3, as well as in miR-146a, miR-182 and miR-200b. Incubation of cortical neural cultures with these ethanol-treated astrocyte-derived EVs increased the expression of inflammatory proteins (e.g., COX-2) and miRNAs (e.g., miR-146a). miR-146a expression is involved in the regulation of genes related with inflammatory pathways. Through Toll-like receptor 4 (TLR4) activation, the astrocyte-derived EVs were able to transmit and trigger inflammation signaling induced by ethanol exposure [46].

Crenshaw et al. investigated the effects of alcohol exposure on the biogenesis and composition of microglia BV-2 cell line-derived exosomes. In addition to the observed decrease in cell viability [47], ethanol exposure significantly decreased CD18, a microglial and immune cell marker, in BV-2 derived exosomes. Furthermore, both heat shock proteins Hsp70 and Hsp90 were increased, suggesting a role in pro-inflammatory responses through ligation of the Toll-like receptors of immune cells. Finally, ethanol administration to BV-2 cells also caused decreased expression of Rab 7 protein, which plays an important role in vesicle trafficking and exosome biogenesis [47].

Exosomes can also provide biological information that could be used for the early diagnosis of fetal neurodevelopmental-related outcomes. Recently, a method for diagnosing fetal alcohol syndrome (FAS) was patented, based on identification of fetal neural exosome biomarkers isolated from maternal plasma [54]. The fetal diagnosis should be positive when the levels of the biomarkers HSF1, Bcl-XL, REST, synaptophysin, synaptotagmin, synaptopodin and GAP–43 are statistically significantly lower when compared with a healthy control. Therefore, the fetal neural exosomes isolated from neonatal plasma are useful in diagnosing neonatal neurodevelopmental outcomes.

Non-coding RNAs, both intracellular and extracellular, and miRNA have been found to be altered in FASDs, indicating consequences for normal neuronal development. In addition, these RNAs can also be useful biomarkers of prenatal alcohol exposure and the efficacy of therapeutic strategies [98]. miRNA-9 and miRNA-153, which are known for their relevant role during brain development, are strongly altered upon alcohol exposure. Zebrafish embryos were exposed to ethanol during gastrulation, resulting in a transient suppression of miRNA-9 during the period associated with neural tube closure and the neural crest migration process [99]. Additionally, ethanol was demonstrated to disrupt miR-9 function and its capacity to target gene expression, while miR-9 knockdown recapitulated the morphological defects observed in FASDs, such as microcephaly. miR-153 is another miRNA that was shown to be an important mediator of ethanol teratogenesis and also a conserved miRNA enriched in brain development [100]. Following ethanol exposure, miR-153 was significantly decreased in fetal cortical neural stem cells (NSCs) [101]. In addition, miR-153 has been shown to target the nuclear factor 1 family of transcription factors, NFIA and NFIB, which are essential for neurogenesis and gliogenesis. The previously described transcripts were also seen to be upregulated after ethanol exposure, possibly due to the decrease of miR-153, which, in turn, supports the hypothesis that ethanol affects the developing cortex by interfering in early maturation of NSCs. Furthermore, an in vivo model of developing zebrafish demonstrated that miR-153 levels decreased after ethanol exposure, consequently revealing impaired neurobehavioral development [102]. In vitro cultured NSCs were also used to understand the role of EVs in NSC development and differentiation during ethanol exposure [48]. In these studies, miR-140-3p was identified as another important miRNA affected by ethanol treatment, indicating that ethanol influences the expression of key differentiation-associated mRNA transcripts. In fact, miR-140-3p overexpression favors the accumulation of glial fibrillary acidic protein (GFAP) and a reduction of glutamate aspartate transporter (GLAST) glial progenitors, which is consistent with the observed inhibition of neurogenesis caused by ethanol and the deficits in neuronal maturation observed in FASDs [48].

### 3.5. Acute Bilirubin Encephalopathy

Neonatal hyperbilirubinemia is a severe developmental pathology caused by bilirubin crossing the BBB and accumulating in the brain stem nuclei, cerebellum and basal ganglia [103,104]. Although the genetic association is still not clear, the neurocognitive and CNS developmental deficits may be mediated by bilirubin-induced neuroinflammation [105,106] and apoptosis of neuronal cells [107]. The role of EVs in the pathogenesis of acute bilirubin encephalopathy (ABE) has not been reported to date. However, a recent study addressed the biomarker potency of EVs in ABE. Proteomic profiling of EVs isolated from the CSF of ABE patients allowed the identification of proteins and signaling pathways that are affected in the CNS by bilirubin toxicity [49]. Gene Ontology (GO) annotation analysis provided clues about the link between EVs and the immune-inflammatory response in ABE. The differentially expressed proteins observed in patient exosomes were serum amyloid A-1 protein (SAA1), APP, lipopolysaccharide-binding protein (LBP), C-reactive protein (CRP), immunoglobulin, complement components (C4B and C5), S100 calcium binding protein A9 (S100A9), S100 calcium binding protein A7 (S100A7), defensin alpha 1 (DEFA1) and lactotransferrin (LTF). These proteins are almost all associated with an immune-inflammatory response. The EV cargo proteins S100A9, S100A7, DEFA1 and LTF were altered in ABE and were linked to nerve cell adaptation to hyperbilirubinemia. This indicates that EVs can be used as biomarkers for the early diagnosis of ABE patients. Moreover, the complement proteins C4B and C5 were upregulated in EVs in ABE. It is hypothesized that these are synthetized by neurons and glial cells in order to restore brain homeostasis, neural development and CNS repair [49]. Further studies are needed to explore the potency of EVs for diagnosing ABE in the early onset of the disease. In addition, further studies are required to elucidate the role of EVs in ABE pathogenesis and to understand whether EVs provide a sensible therapeutic strategy to slow down and reverse ABE.

## 4. EVs in Therapy

Our increased understanding of EV biology has opened novel ways for treating diseases, including CNS developmental disorders. The ability of EVs to cross the BBB has contributed to exploring the therapeutic potential of EVs in brain diseases more intensely [108]. Furthermore, the perception that EVs can be engineered and produced with a specific molecular cargo has propelled research into therapeutic applications of EVs [109,110]. The therapeutic potential of EVs in the field of neurodegenerative diseases has been recently reviewed [111]. Reports on therapeutic applications of EVs in neurodevelopmental pathologies are sparse so far. Only recently, and as previously mentioned, exosomes isolated from adipose-derived MSCs were intranasally administrated into different autistic mice models, with improvements in the ADS symptomatology [51]. However, before the development of any clinical application, the cargo loading and the mechanisms of action must be well defined. Moreover, the precise extraction, the yield of production and the molecular characterization of, for example, MSC-derived vesicles should be addressed, since they could vary between different cell sources [112]. Additionally, the optimal therapeutic administration and side effects must be carefully evaluated before approval.

## 5. Conclusions/Final Remarks

A growing body of scientific evidence provides valuable knowledge and understanding of the role of EVs during CNS development in health and disease. Our literature review indicates the pertinent role of EVs in various CNS disorders. Although the pathways in which EVs, mainly exosomes, are involved have been identified and EV cargo has been linked to cellular responses, further examinations are required to grasp a full understanding of the role of EVs in the dynamics of the CNS. Such studies will also strengthen the basis for utilization of EVs in diagnosis and treatment of CNS disorders. Interestingly, the distinctive feature of EVs, particularly exosomes, of crossing the BBB provides a tremendous advantage in designing EV based diagnostics and therapeutics for CNS disorders. Recent developments in the field of exosome engineering [110] will further catalyze the development of EV-based therapeutics. These technologies make it possible to generate exosomes customized for a specific CNS pathology. Future clinical studies should demonstrate the clinical benefits of the exosome-based diagnostic and therapeutic avenue.

## Figures and Tables

**Table 1:** EV cargo alterations associated with various models of human neurodevelopmental disorders.

Disease	EVs—Type and Source	EVs—Cargo Alterations in Disease Context	Reference
Rett syndrome (RTT)	Exosomes from hiPSC-derived neurons (from both isogenic control and MeCP2LOF-disesase cell lines)	Proteomic analysis revealed a downregulation in neurodevelopmental signaling proteins associated with neuronal maturation, axonal guidance and synaptogenesis.	[39]
Autism spectrum disorder (ASD)	EVs isolated from ASD children’s serum	Significant increase in total protein concentration and in the amount of mtDNA in EVs.	[40]
Down syndrome (DS)	Exosomes from a Ts2 mice model with DS-like phenotype	Increased levels of exosomes in DS models influenced by larger and more abundant number of MVBs and more ILVs per neuron; Neuronal exosomes with a homeostatic role for neurotoxic material release in response to chronic endosomal dysfunction.	[41]
	Exosomes isolated from DS patients, Ts2 mouse brains and human DS fibroblasts	Enriched in APP carboxyl-terminal fragments (APP-CTFs) and in full-length APP (flAPP).	[42]
	Exosomes from blood samples from DS patients	Increased CD81 levels (more abundant neuronal exosomes secreted). Neuronal exosomes contained Aβ peptide products and hyper-phosphorylated species of Tau (P-Tau).	[43,44]
	Exosomes from blood samples of DS patients	DS neuronal exosomes showed higher levels of Aβ1-42, phosphorylated P-T181-Tau and P-S396-Tau.	[45]
Fetal alcohol syndrome (FAS)	EVs from cultured neurons and astrocytes (ethanol-treated)	EVs from the treated condition are increased in number, with higher content of inflammatory-related proteins, such as TLR4, NFκB-p65, IL-1R, caspase-1 and NLRP3, as well as miRNAs (miR-146a, miR-182 and miR-200b).	[46]
	Exosomes from microglia BV-2 cell lines (exposed to ethanol during biogenesis)	Decreased levels of CD18 (a microglial and immune cell marker). Both Hsp70 and Hsp90 were increased (preventing damaging pro-inflammatory responses). Decreased expression of Rab 7 protein, (important role in vesicle trafficking and exosome biogenesis).	[47]
	EVs from an in vitro model of NSCs (exposed to ethanol)	miR-140-3p was identified to be increased during ethanol treatment, which could influence neurogenesis inhibition and neuronal alterations.	[48]
Acute bilirubin encephalopathy (ABE)	EVs isolated from the CSF of ABE patients	Differentially expressed proteins associated with immune-inflammatory response, such as SAA1, APP, LBP, CRP, immunoglobulin and complement components (C4B and C5). Altered levels of specific EVs cargo, mainly S100A9, S100A7, DEFA1 and LTF.	[49]

**Table 2:** EVs cultured or administrated, and their therapeutic effect.

Disease	EVs—Type and Source	EVs—Culture/Administration	EVs—Therapeutic Effect	Reference
Rett syndrome (RTT)	Exosomes extracted from IC hiPSC-derived neurons	Exosomes cultured with MeCP2LOF hiPSC-derived neurons	Increased/improved: Puncta densities; Synaptogenesis; Neuronal activity (higher network synchronization); Proliferation; Neuronal fate in developing neural cultures.	[39]
Autism spectrum disorder (ASD)	EVs isolated from ASD children’s serum	EVs cultured with human microglia cells	Increased Secretion of IL-1β, a pro-inflammatory cytokine.	[40]
	Exosomes secreted by mesenchymal stem cells (MSCs)	Exosomes were intranasally administrated in a BTBR mouse model (presents autistic-like behaviors and ASD symptoms)	Increased: Male to male social interaction; Reduced: Repetitive behaviors; miRNA-143 cargo (an immunomodulatory effector in the host cells).	[50]
	Exosomes from adipose-derived MSCs	Exosomes were intranasally and intravenously administrated into BTBR and Shank3 mice models (ASD cognitive and motor impairments)	Improved: ASD behavioral phenotype (mainly by non-invasive intranasal administration).	[51]
	Exosomes from adipose-derived MSCs	Exosomes cultured with primary neuronal cell cultures, prepared from a newborn SHANK3 homozygote mouse model of autism.	Upregulated: Proteins related to anti-inflammatory processes; Proteins related to immunomodulation; BDNF (neuroprotection and neurogenesis mediator).	[52]
Down syndrome (DS)	Neuronal-derived exosomes purified from the blood of individuals with DS-AD and controls	Exosomes were injected into a control mouse model	Increased: P-Tau amount in pyramidal neurons and in the dentate gyrus of the hippocampus; Spread of toxic P-Tau species via exosome mediation (unpublished work)	[53]
Fetal alcohol syndrome (FAS)	EVs from cultured neurons and astrocytes (ethanol-treated)	Ethanol-treated EVs from astrocytes were incubated with cortical neural cultures	Increased: Levels of the inflammatory protein COX-2; miRNAs: miR-146a (regulation of genes related to inflammatory pathways)	[46]

**Table 3:** EVs as potential biomarkers for neurodevelopment-related disorders.

Disease	EVs—Type and Source	EVs—Biomarker Potential	Reference
Down syndrome (DS)	Exosomes from DS patients’ blood samples	Increased levels of: CD81; Aβ peptide products; Hyper-phosphorylated species of Tau (P-Tau)	[43,44]
	Neural origin exosomes extracted from DS patients’ blood samples	Elevated levels of: Aβ1-42; Phosphorylated P-T181-Tau; P-S396-Tau	[45]
Fetal alcohol syndrome (FAS)	Fetal neural exosomes isolated from maternal plasma	Significantly lower levels of: HSF1; Bcl–XL; REST; Synaptophysin; Synaptotagmin; Synaptopodin; GAP–43	[54]
Acute bilirubin encephalopathy (ABE)	EVs isolated from the CSF of ABE patients	Upregulated: LTF and DEFA1 C4B and C5 (complement-associated proteins) Downregulated: S100A7 and S100A9	[49]

## Abbreviations

ABE	acute bilirubin encephalopathy
ASD	autism spectrum disorder
AD	Alzheimer’s disease
AMPA	2-amino-3-(3-hydroxy-5-methyl-isoxazol-4-yl) propanoic acid
Aβ	β-amyloid
APP	β-amyloid precursor protein
APP-CTFs	APP carboxyl-terminal fragments
GABA	gamma-aminobutyric acid
BBB	blood–brain barrier
BDNF	brain-derived neurotrophic factor
CNS	central nervous system
CSF	cerebrospinal fluid
DS	Down syndrome
DYRK1A	dual-specificity tyrosine-phosphorylation-regulated kinase 1A
E/I	excitatory/inhibitory imbalance
ESCRT	endosomal sorting complex required for transport
FAS	fetal alcohol syndrome
FASDs	fetal alcohol spectrum disorders
EVs	extracellular vesicles
flAPP	full-length APP
GAPDH	glyceraldehyde-3-phosphate dehydrogenase
GLAST	glutamate aspartate transporter
GLT1	glucose transporter type 1
GFAP	glial fibrillary acidic protein
GluR2/3	glutamate receptor subunit
GPI	glycosylphosphatidylinositol
hiPSCs	human induced pluripotent stem cells
Hsa21	chromosome 21
Hsp	heat-shock proteins
IC	isogenic control
IL	interleukin
ILVs	intraluminal vesicles
L1CAM L1	cell adhesion molecule
LPS	lipopolysaccharides
MeCP2	methyl-CpG-binding protein 2
MeCP2LOF	methyl-CpG-binding protein 2 loss of function
miRNAs	micro RNAs
mRNAs	messenger RNA
MSCs	mesenchymal stem cells
mtDNA	mitochondrial DNA
MVs	microvesicles
MVBs	multivesicular bodies
NPCs	neural progenitor cells
NMDA	N-methyl-d-aspartate
RG	radial glia
RNA	ribonucleic acid
ROS	reactive oxygen species
RTT	Rett syndrome
siRNA	small interfering RNA
TGF- β1	anti-inflammatory cytokine tumor growth factor–β1/transforming growth factor beta 1
TLR4	toll-like receptor 4
TrkB	tropomyosin-related kinase B
